# Detection of *bla*OXA-145, *bla*OXA-224, *bla*OXA-539, and *bla*OXA-675 Genes and Carbapenem-Hydrolyzing Class D *β*-Lactamases (CHDLs) in Clinical Isolates of *Pseudomonas aeruginosa* Collected from West of Iran, Hamadan

**DOI:** 10.1155/2022/3841161

**Published:** 2022-08-05

**Authors:** Arash Sezadehghani, Sanaz Dehbashi, Hamed Tahmasebi, Mohammad Reza Arabestani

**Affiliations:** ^1^Microbiology Research Center, Pasteur Institute of Iran, Tehran, Iran; ^2^Department of Laboratory Sciences, Varastegan Institute of Medical Sciences, Mashhad, Iran; ^3^School of Medicine, Shahroud University of Medical Sciences, Shahroud, Iran; ^4^Department of Microbiology, Hamadan University of Medical Sciences, Hamadan, Iran; ^5^Brucellosis Research Center, Hamadan University of Medical Sciences, Hamadan, Iran

## Abstract

Carbapenem-hydrolyzing class D *β*-lactamases (CHDLs) are on the rise and are a major public health problem worldwide. *Pseudomonas aeruginosa* is resistant to carbapenem; currently, the most effective treatment option is being increasingly reported. This study aimed to identify *bla*OXA-145, *bla*OXA-224, *bla*OXA-539, and *bla*OXA-675 genes in CHDL strains. Samples were collected from clinical specimens admitted to the hospital. Antibiotic susceptibility was determined using the disk diffusion methods. CHDL strains were detected using a phenotypic method (disk diffusion). The PCR method was used to identify *bla*OXA-145, *bla*OXA-224, *bla*OXA-539, and *bla*OXA-675 genes. Piperacillin-resistant strains (*n* = 9, 17.4%) had the lowest frequency, and cefoxitin-resistant strains (*n* = 100, 91.7%) had the highest distribution in *P. aeruginosa* isolates. Also, 29.35%, 12.8%, and 8.2% were multidrug-resistant, extensively drug-resistant, and pan drug-resistant, respectively. MBL-producing *P. aeruginosa* and KPC-producing *P. aeruginosa* were detected, respectively, in 47.7% and 37.6% of isolates. Biofilm formation was observed in 63.3% of *P. aeruginosa* isolates. The frequency of OXA genes was as follows: *bla*OXA-145 gene in 30 isolates (27.5%), *bla*OXA-224 in 24 isolates (22.0%), *bla*OXA-539 in 22 isolates (20.1%), and *bla*OXA-675 in 13 isolates (11.9%). However, 19 (17.4%) isolates carry all *bla*OXA-145, *bla*OXA-224, *bla*OXA-539, and *bla*OXA-675 genes. The antimicrobial resistance and OXA genes among biofilm former strains were significantly higher than those of nonbiofilm former strains (*p* < 0.05). The emergence of carbapenem-resistant isolates of *P. aeruginosa* has posed serious threats to the community because they exhibit multiple drug resistance, thus limiting the therapeutic options for clinicians.

## 1. Introductions

Hydrophobic drugs enter bacterial cells through the phospholipid layer, while hydrophilic drugs enter through porins in Gram-negative bacteria (GNB) [1, 2]. Some bacterial species, such as *Pseudomonas aeruginosa*, have an outer membrane that is less porous than other species, causing the bacteria to be less susceptible to antimicrobial agents [3, 4]. *P. aeruginosa* is the most common organism isolated among the nonfermenters from the clinical specimens. Resistance to various antibacterial drugs can be acquired through mutations causing loss, reduced size, or decreased expression of outer membrane proteins (OMPs) in bacteria [3, 5]. Infections caused by bacterial pathogens with multidrug-resistant (MDR), extremely drug-resistant (XDR), and pan drug-resistant phenotypes (PDR) are challenging and difficult to treat. *P. aeruginosa* is resistant to carbapenem; currently, the most effective treatment option is being increasingly reported [6, 7].

Resistance to carbapenems is often mediated by metallo-beta-lactamase (MBL) production. A class B type of beta-lactamases requires divalent metal ions, usually zinc, for their activity [8]. MBL production is a significant problem in hospital isolates of *P. aeruginosa*; the accurate identification and reporting of MBL-producing *P. aeruginosa* will aid infection control practitioners in preventing the spread of these multidrug-resistant isolates [4, 9]. Many phenotyping methods have been performed to search for MBL enzymes of *P. aeruginosa* strains. Chromosomal genes produced all these enzymes and were at first recorded only from single clinical isolates [10]. They may be chromosomal or plasmid-mediated and therefore threaten the spread of resistance by gene transfer among GNB. Since several mechanisms mediate carbapenem resistance, cross-resistance is commonly seen among related antibiotics [11].

*β*-lactamases capable of hydrolyzing third-generation cephalosporins and other narrow-spectrum antibiotics such as penicillin are generally referred to as extended spectrum *β*-lactamases (ESBLs) [10]. There are currently over 200 ESBLs within the molecular classes A, C, and D. Most of these enzymes do not exhibit CPase activity. Carbapenem antibiotics are thus the treatment of choice for infections caused by ESBL-expressing strains [4, 10, 12]. However, this increased use of carbapenems in response to ESBL activity is likely a factor in applying selective pressure for organisms to acquire and express carbapenem hydrolyzing class D *β*-lactamases (CHDLs) [13]. Some OXA-type *β*-lactamases may also hydrolyze carbapenems. However, at least at a significant level, they cannot combine such carbapenem-hydrolyzing activities with traditional ESBL hydrolytic profiles [14, 15].

CHDLs are allocated in the subgroup 2df, more frequent in nonfermenters Gram-negative bacteria (NFGNB). Except for the clinically essential enzymes from *A. baumannii*, OXA-23 and OXA-58 are plasmid-encoded [16]. The CHDLs described so far in this opportunistic pathogen are very likely chromosomally located. These acquired enzymes, OXA-23 and OXA-58, have contributed significantly to carbapenem resistance [15]. Naturally-occurring CHDLs include OXA enzymes such as OXA-24, OXA-51, and OXA-69, all from NFGNB [16]. However, the frequency of *bla*OXA-145, *bla*OXA-224, *bla*OXA-539, and *bla*OXA-675 genes in *P. aeruginosa* in Iran is unknown. Also, the association of these genes with CHDLs is not clear. Therefore, this study aimed to detect *bla*OXA-145, *bla*OXA-224, *bla*OXA-539, and *bla*OXA-675 genes and carbapenem-hydrolyzing class D *β*-lactamases (CHDLs) in clinical isolates of *P. aeruginosa* collected from west of Iran.

## 2. Materials and Methods

### 2.1. Study Design, Isolation, and Identification of *P. aeruginosa*

In this cross-sectional study, 580 clinical samples were collected, including pus, sputum, urine, blood, ascitic fluid, endotracheal fluid, bronchoalveolar lavage, and wound swab, from the patients who were admitted to various clinical departments at Hamadan's hospital. The research was carried out between June 2020 and May 2021. Isolates (one per patient) were obtained from inpatients and outpatients who presented with symptoms of bacterial infections. Ethical committee clearance has been obtained from the institution and written informed consent was received from the patients before collecting the specimens (ethical code number: IR.UMSHA.REC.1398.1007). Identification was mainly based on Gram staining, colony morphology on nutrient agar, MacConkey agar, blood agar, characteristic odor in culture plates, oxidase test, motility, biochemical reactions, and growth at 42°C [4, 17].

### 2.2. Antibiotic Susceptibility Test

Antibiotic susceptibility testing was performed for all the *P. aeruginosa* isolates as per the standard CLSI 2021 guidelines for the following antimicrobials using the Kirby–Bauer disc diffusion method [18]. Piperacillin (75 *μ*g), gentamicin (10 *μ*g), amikacin (30 *μ*g), ciprofloxacin (5 *μ*g), ceftazidime (30 *μ*g), ceftriaxone (30 *μ*g), imipenem (10 *μ*g), meropenem (10 *μ*g), erythromycin (10 *μ*g), cefoxitin (30 *μ*g), cefazolin (10 *μ*g), and cefepime (30 *μ*g). All discs belonged to MAST, UK. The control strain used was *P. aeruginosa* ATCC 27853. An overnight broth culture compared to 0.5 McFarland's was used as the inoculum. After incubation at 37°C for 16–18 hrs, a zone of inhibition was noted. Multidrug-resistant (MDR) isolates were estimated to be resistant to three or more drugs of therapeutic relevance [18].

### 2.3. Screening of Biofilm-Forming Isolates

Microtiter plate assay with crystal violet was used for testing the biofilm formation ability of different *P. aeruginosa* isolates. The culture was then diluted to 1 : 100 into a fresh medium (BHI) for biofilm assays. 100 *μ*L of the dilution was added per well in a 96-well dish. The microtiter plate was incubated for 24 hrs at 37°C. After incubation, the cells were dumped out by turning the plate over and shaking the liquid. Gently, the plate was submerged in a small tub of water. Excess water was shacked. This process was repeated for the second time. Then, 125 *μ*L of a 0.1% solution of crystal violet (CV) in water was added to each well of the microtiter plate. The microtiter plate was incubated at room temperature for 10–15 mins. As outlined, the plate was rinsed 3–4 times with water by submerging in a tub of water. The microtiter plate was turned upside down and dried overnight. The wells were photographed when dry for qualitative analysis. For quantitative analysis, 125 *μ*L of 30% acetic acid in water was added to each microtiter plate well to solubilize the CV. The microtiter plate was incubated at room temperature for 10–15 mins. 125 *μ*L of the solubilized CV was transferred to a new flat-bottomed microtiter dish. The absorption of the eluted stain was measured at 590 nm using an ELISA reader. The classification of biofilm formation was determined based on the following formula: less than 0.120: nonbiofilm producer; in the range of 0.120–0.240: moderate biofilm producer; greater than 0.240: strong biofilm producer [19].

### 2.4. Screening of KPC-Producing Isolates by the Modified Hodge Test

The indicator bacterium, *Escherichia coli* ATCC 25922, was used to inoculate a Mueller–Hinton agar plate with zinc sulfate at a 70 gm/ml turbidity of 0.5 McFarland standard. The test strain was heavily streaked from the center to the plate periphery. A 10 *μ*g imipenem disk was placed in the center of the plate. After the plate had been left at room temperature for 15 minutes. The plate was incubated overnight. A distorted inhibition zone was a positive result for carbapenem hydrolysis screening. *Klebsiella pneumonia* ATCC 70063 was used as a positive control, and *P. aeruginosa* PAO-1 was used as a negative control [4, 19].

### 2.5. Screening of MBL-Producing Isolates by the Combine Disc Test (CDT)

All imipenem-resistant *P. aeruginosa* isolates (Imipenem (10 *μ*g) zone ≤15 mm) were tested for the production of the MBL enzyme. The Mueller–Hinton agar plates were inoculated with 0.5 McFarland bacterial suspension of the test organism. Imipenem (10 *μ*g) and imipenem-EDTA (10/750 *μ*g) disks were placed 30 mm apart from each other. The inhibition zones of the imipenem and imipenem-EDTA disks were compared. An increase in the zone diameter ≥7 mm indicated the presence of metallo-*β*-lactamase. Plates were incubated at 35 degrees Celsius for 24 hours. The zone of inhibition was compared with each other. *P. aeruginosa* ATCC 15442 was used as a positive control, and *P. aeruginosa* PAO-1 was used as a negative control [1, 4].

### 2.6. DNA Extraction and Polymerase Chain Reaction (PCR)

DNA was extracted using a CinaClone bacterial genomic DNA purification kit (QIAamp DNA mini kit; Qiagen, Hilden, Germany). The concentration of DNA extracted from each sample was measured using a NanoDrop ONE (Thermo Fisher Scientific, Wilmington, DE, USA) and then immediately subjected to PCR. The primers were obtained from CinaClone Biotech Services Private Limited, Tehran (Table 1). The PCR was performed by the conventional method using an Eppendorf thermal cycler under the following conditions: each single reaction mixture (25 *μ*l) contained 4 *μ*l of DNA suspension, 12.5 *μ*L of master mix (10 mM dNTPs, 1 U Taq DNA polymerase, 25 mM MgCl_2_, and 2.5 *μ*l of 10× Taq buffer) and 1 *μ*M of each primer (Sigma-Aldrich, Mumbai). The remaining volume was adjusted with PCR grade water with initial denaturation at 95°C for 2 minutes, 32 cycles of 95°C for 25 seconds, annealing temperature at 60°C for 45 seconds, an extension at 72°C for 1 minute, and a final elongation at 72°C for 3 minutes. Agarose gel electrophoresis was performed with agarose, 50X TAE buffer, 6X gel loading buffer, and SYBR® safe DNA gel stain. The size of the DNA band was analyzed using a UV transilluminator and photographed using an AlphaImager mini analysis system (Alpha Innotech-ProteinSimple, San Jose, CA, USA). *P. aeruginosa* ATCC 15442 was used as a positive control, and *P. aeruginosa* PAO-1 was used as a negative control.

### 2.7. Statistical Analysis

Results were analyzed to determine if there was any significant difference between carbapenem susceptibility and nonsusceptibility among individual antibiotics. A nonparametric analysis (i.e., Fisher's exact test) was used for our data. All analyses were conducted using GraphPad Prism (version 8.0; California USA). The chi-square test was applied to see the significance of two categorical variables. *P*-value <0.05 is considered statistically significant.

## 3. Results

In the current study, 109 *P. aeruginosa* were isolated from different clinical samples. According to Table 2, most samples were obtained from 35 urine samples (32.1%), followed by 29 blood samples (26.6%), 19 wound samples (17.4%), 15 sputum samples (13.7%), and 11 body fluid samples (10.1%).

### 3.1. Antibiotic Susceptibility Test

**Figure 1:**
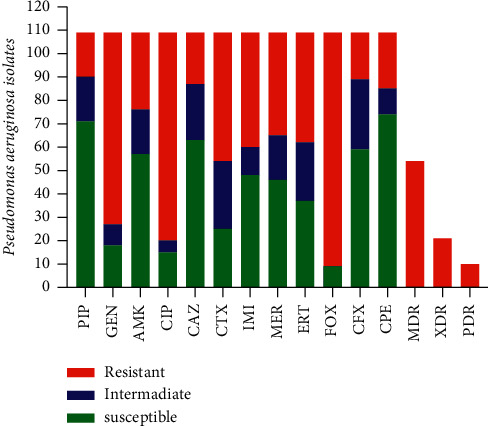
Antibiotic resistance pattern in clinical isolates of *P. aeruginosa*. PIP: piperacillin; GEN: gentamycin; AMK: amikacin; CIP: ciprofloxacin; CAZ: ceftazidime; CTX: ceftriaxone; IMI: imipenem; MER: meropenem; ERT: ertapenem; FOX: cefoxitin; CFX: cefazolin; CPE: cefepime; MDR: multiple drug resistance; XDR: extensively drug-resistant; PDR: pan drug-resistant.

According to Figure 1, *P. aeruginosa* isolates show the highest resistance to cefoxitin (91.7%), ciprofloxacin (81.6%), gentamicin (75.2%), and ceftriaxone (50.4%). Also, among the 109 clinical isolates of *P. aeruginosa,* 32 (29.35%) were MDR, 14 (12.8%) were XDR, and 9 (8.2%) were PDR.

### 3.2. Prevalence of Biofilm-Forming Isolates

Out of the 109 isolates shown in Table 3, 17 isolates (15.5%) produced a strong biofilm, 41 (37.6%) produced a moderate biofilm, and 11 isolates (10.1%) produced a weak biofilm. The remaining 40 isolates (36.6%) did not have any biofilm.

### 3.3. Prevalence of MBL-Producing *P. aeruginosa*

**Figure 2:**
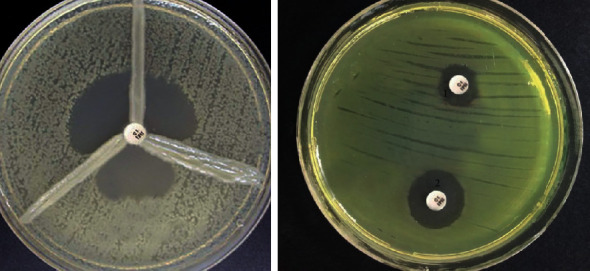
The result of phenotypic detection of MBL-producing *Pseudomonas aeruginosa* by the EDTA-imipenem microbiological (EIM) test in *Pseudomonas aeruginosa*. (a) EDTA + imipenem; (b) imipenem. Top: MBL positive strain; bottom: MBL negative strain; MBL considered positive when the zone diameter difference between imipenem + EDTA and imipenem discs was larger than 7 mm (right). The result of phenotypic detection of carbapenemase-producing *P. aeruginosa* by the modified Hodge test (left).

Table 2 and Figure 2 show that the overall prevalence of MBL-producing *P. aeruginosa* was 37.6% (*n* = 41). For result interpretation, we use this result as the CLSI recommends this technique as a reference for other phenotypic methods.

### 3.4. Prevalence of KPC-Producing *P. aeruginosa*

In Table 2 and Figure 2, 47.7% of isolates (52) of *P. aeruginosa* were phenotypically confirmed for MBL using the combination disk method.

### 3.5. The Prevalence of OXA Genes

**Figure 3:**
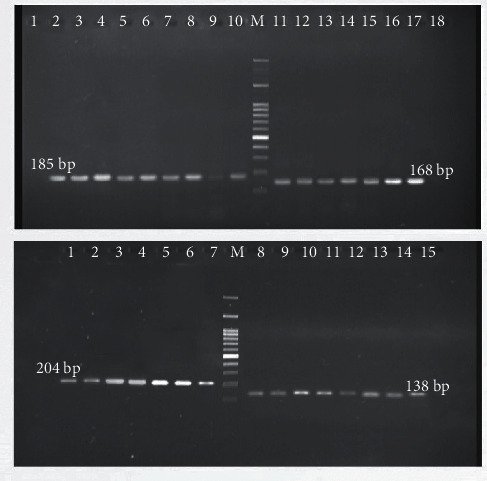
The amplification and gel electrophoresis agarose of 1.5% of *bla*OXA genes in *P. aeruginosa*. Top: *bla*OXA-539 with 185 bp and *bla*OXA-224 with 168 bp; wells 1 and 17: positive control, well 18: negative control, wells 3–9: positive strains with *bla*OXA-539; wells 11–18: positive strains with *bla*OXA-224. Down: *bla*OXA-145 with 204 bp and *bla*OXA-675 with 138 bp; wells 1 and 14: positive control, well 15: negative control wells 2–7: positive strains with *bla*OXA-145, wells 8–14: positive strains with *bla*OXA-145. M: ladder 100 bp.

Based on Table 3 and Figure 3, out of 109 *P. aeruginosa*, 13 (11.9%) isolates carry all *bla*OXA-145, *bla*OXA-224, *bla*OXA-539, and *bla*OXA-675 genes. However, *bla*OXA-145 gene in 20 isolates (18.3%), *bla*OXA-224 in 24 isolates (22.0%), *bla*OXA-539 in 22 isolates (20.1%), and *bla*OXA-675 in 13 isolates (11.9%) were detected.

## 4. Discussions

Two hundred isolates of *P. aeruginosa* were taken from various clinical samples such as wound (17.4%), urine (32.1%), blood (26.6%), sputum (13.7%), and body fluids (10.1%) for their role in infections in hospitalized patients including the characteristics of their drug resistance.

This is in accordance with the study of Tahmasebi et al. [20]., who have also reported the isolation of *P. aeruginosa* to be higher in urine samples followed by blood samples and wound samples. This is in contrast to the study conducted by Zahedani et al. [19]., who reported the highest occurrence of *P. aeruginosa* isolates in wound and blood samples.

The antibiotic sensitivity results showed a high sensitivity report to many antibiotics used. *P. aeruginosa* isolates showed the highest sensitivity to piperacillin (82.5%), followed by cefazolin (81.6%) and ceftazidime (79.8%), as shown in Table 2. However, in our study, increased resistance to cefoxitin (91.7%) and ciprofloxacin (81.6%) was observed, which was similar to the observation made by Zahedani et al. [19], who reported ciprofloxacin resistance to be around 69.6%. The antibiotic susceptibility testing also noted high resistance to antibiotics such as gentamicin (75.2%) and ceftriaxone (50.4%). As in the current study, numerous other researchers have already reported a decreased susceptibility of *P. aeruginosa* to the commonly used antibiotics (Dehbashi et al. [10] and Adekunle et al. [21]). Among *P. aeruginosa*, 49.5% of isolates were MDR, 19.2% were XDR, and 9.1% were PDR. This is higher than those reported in the studies in Iraq [22] and Spain [23]. Nevertheless, comparable to the survey by El-Baky et al., the study in [24] showed that >70% of *P. aeruginosa* isolates were MDR and XDR. They used the Kirby–Bauer method to analyze the susceptibility of the isolates for interpretation. Yet, they found different estimates suggesting true differences in the prevalence of resistant isolates in the study populations.

In the present study, 40.3%, 43.1%, and 44.9% of isolates, respectively, were resistant to meropenem, ertapenem, and imipenem. Carbapenemases are *β*-lactamases, which include serine-*β*-lactamases (KPC, OXA, and GES genes) and metallo-*β*-lactamases (MBLs). Whereas, in the study performed by Tahmasebi et al. [8]., a high rate of ertapenem-resistant and meropenem-resistant strains among the total isolates are shown. By contrast, some studies in China [25] and Chile [26] used the *E*-test method and clinical breakpoints to determine the resistance of *P. aeruginosa* isolates, so that differences in prevalence estimates between their study and the current study may partly be due to differences in methodology.

In our reports, MBL- and KPC-producing strains were detected in 47.7% and 37.6% of *P. aeruginosa* isolates, respectively. This argument is consistent with the findings of Yoon and Jeong [26], which contrasts with the study performed by Shahin and Ahmadi which reported a high frequency of MBL- and KPC-producing *P. aeruginosa* [12]. This difference could be due to the difference in the study environment under which the study was performed. Also, these findings are an alarming indication that an outbreak of MBL- and KPC-producing strains can occur in different hospitals that provide various services to patients: maternity, pediatric, surgical, and general hospitals.

A moderate number of resistant isolates and MBL- and KPC-producing strains have been obtained from wound samples of burn patients. *P. aeruginosa* is the second most common organism causing infections in burn patients. Burns cause a skin barrier breach, creating complimentary access for organisms causing infections. Therefore, *P. aeruginosa* can easily colonize a burn victim because of its saprophytic nature. Mesbahi et al. [11] and Tahmasebi et al. [8] state that the organism causing infection in a burn patient and its susceptibility should be known to select the appropriate antibiotics to prevent the emergence of resistance and its spread. Poor infection control practices are found to be an essential causation factor for burn wound infections. This shows that the estimates of antimicrobial resistance prevalence and public health concerns can be highly dependent on the choice of the threshold.

Based on the current study, 15.5% of *P. aeruginosa* isolates produced a strong biofilm, 37.6% produced a moderate biofilm, and 10.1% of isolates produced a weak biofilm. In this study, biofilm moderate biofilm producers and strong biofilm producers have shown equal resistance to all groups of drugs. Although, the survey conducted by Lima et al. [27] stated that nonproducers had shown increased resistance compared to strong biofilm producers. The same findings were also noted in the study conducted by Kamali et al.[28], who documented that 83.7% of isolates of *P. aeruginosa* were biofilm producers. Lima et al. [27]. reported that 47.5% of *P. aeruginosa* were slime producers.

In the present study, we reported a significant association between biofilm formation and increased prevalence of antibiotic resistance (*P*=0.001). Within a biofilm, bacteria can be protected by the exopolysaccharides inhibiting the entry of antimicrobial compounds either through the thickness of the biofilm or by causing compounds to bind to the matrix. Additionally, bacteria embedded in a biofilm have a lower metabolic activity which slows their uptake of antimicrobial compounds, making the antibiotic ineffective. In addition, the colonies of the organism form biofilms within which they are protected from the host's defenses and antimicrobial agents, and communicate with each other through the complex cell-to-cell signaling called quorum sensing. Nevertheless, Zahedani et al. [19] observed that most biofilm producer *P. aeruginosa* isolates were MDR. Tahmasebi et al. [8] found that biofilm generation was considerably greater in MDR isolates in another research.

In this study, *bla*OXA-145, *bla*OXA-224, *bla*OXA-539, and *bla*OXA-675 genes were detected in 18.3%, 22.0%, 40.3%, and 10.1% of *P. aeruginosa* isolates, respectively. This is one of the most widespread genes encoding for resistance. No studies to detect these genes in Iran isolates have been carried out. Nevertheless, a high abundance of *bla*OXA-224 and *bla*OXA-539 genes was observed in KPC- and MBL-producing *P. aeruginosa*. *bla*OXA-224 and *bla*OXA-539 carbapenemase-producing clones have led to a recent increase in carbapenem resistance. This supports our study's higher incidence of blaOXA-224- and blaOXA-539-like carbapenemases. The *bla*OXA-224- and *bla*OXA-539-like and coproducers of both are the common genes isolated in carbapenem-resistant isolates in a study conducted by Ribot et al. [29].

Carbapenem resistance is predominantly due to *bla*OXA-675-like enzymes that are hard to see in KPC-producing *P. aeruginosa*. In our study, two KPC-producing *P. aeruginosa* isolates harbored the *bla*OXA-675 gene. This is in agreement with the study conducted by Delgadoa et al. [30], who reported a low prevalence of the OXA-1-like beta-lactamase family genes in KPC- and MBL-producing *P. aeruginosa*. Nonetheless, the OXA-2 family metallo-*β*-lactamase is the prevalent gene in studies conducted in Spain [31] and Sweden [32]. The MBL- and KPC-producing bacteria are considered the most frequent cause of urinary tract infections. This fact supports our finding of more carbapenem-resistant isolates obtained from urine samples in this study. They are also found to cause bloodstream infections and wound infections. Bacteria having MBL can spread rapidly (horizontal MBL gene transfer) within the hospital environment and across continents, posing therapeutic and control management problems. Molecular detection was performed for the KPC- and MBL-producing isolates where OXA genes were not detected for some isolates. This could be explained by the fact that the isolates show MBL resistance by the presence of genes other than blaIMP and blaVIM, which needs further evaluation.

Ahmed et al. [32] stated that *bla*OXA-224 and *bla*OXA-539 belong to the OXA-2 family and are among the most important genes for resistance to carbapenems and ceftazidime in multidrug-resistant *P. aeruginosa*. They also demonstrated that the mutant OXA-4 and the selection for extended-spectrum *bla*OXA-539 result in high-frequency antibiotic resistance in XDR and PDR strains.

The correlation between biofilm formation and OXA genes in the present study was consistent with the findings of Park and Koo [33]. More than 60% of the biofilm-producing strains carried the *bla*OXA-145, *bla*OXA-224, *bla*OXA-539, and *bla*OXA-675 genes. Nevertheless, some studies showed no correlation between *bla*PER-1-positive isolates and biofilm producers [34]. Therefore, it is considered that the presence of OXA genes is more crucial for cell adhesion than biofilm formation. The reasons for this have not yet been fully explored since no data is available on the knockout of *bla*OXA-145, *bla*OXA-224, *bla*OXA-539, and *bla*OXA-675 genes in *P. aeruginosa*.

The data thus obtained by our study reiterates the importance of *P. aeruginosa* as an invasive pathogen and is aimed at tracking their occurrence and designing means of reducing their impact. Hence, it is high time we realized the threat posed by carbapenem resistance in *P. aeruginosa* isolates and took measures to control the spread of these organisms. Although there is evidence of a causal link between antibiotic consumption and resistance, it remains a complex issue. Recently, there has been a focus on antimicrobial consumption and resistance patterns to understand the local epidemiology in formulating a hospital antibiotic policy.

## 5. Conclusion

The study results conclude that *P. aeruginosa* is a critical opportunistic pathogen and is resistant to commonly used antibiotics. It also emphasizes the importance of speciation of *P. aeruginosa* and knowing carbapenems' resistance in the isolates. The prevalence of pathogens often varies between hospitals and patients in the same hospital. Clinical microbiologists must report and update clinicians on the circulating pathogens' prevalence and antimicrobial susceptibility pattern. The antibiotics to be used for empiric therapy should be selected accordingly.

However, judicious use of antibiotics, rapid isolation of patients suspected to have carbapenem-resistant *Pseudomonas aeruginosa* infections, and regular testing of all isolates for metallo-beta-lactamase production among *Pseudomonas aeruginosa* are recommended for the prevention of remission of carbapenem-resistant *P. aeruginosa*.

## Figures and Tables

**Table 1:** Oligonucleotide sequences used in this study and thermal cycling conditions.

Gene	Sequence of primers	Thermal cycles	Product size (bp)	Ref
*bla*OXA-145	F: CAAATGGGACGGAAAGCCAA R: AGCTGACCCTCCCAGAATTT	95°C/5 min; (95°C/1 min, 60°C/30sec, 72°C/45 sec) X30; 72°C/5 min	204	This study

*bla*OXA-224	F: AGTGTGACGGAATCGTTGCT R: GGCGCGGCTTAACTCAAGCGT	95°C/5 min; (95°C/1 min, 61°C/1 min, 72°C/45 sec) X30; 72°C/5 min	168	This study

*bla*OXA-539	F: TGATGCACTGGCGCTGCTGT R: GATTTTTCGATGGGACGGCG	95°C/5 min; (95°C/1 min, 60°C/45 sec, 72°C/1 min) X30; 72°C/5 min	185	This study

*bla*OXA-675	F: TCAGCATCAAAAGAAAATCAGC R: ATGATTTTGGTGGGAATGGA	95°C/7 min; (95°C/1 min, 60°C/1 min, 72°C/50 sec) X35; 72°C/10 min	138	This study

**Table 2:** Carbapenemase-producing and biofilm-forming capacity of *P. aeruginosa,* and percentages of their OXA genes related to antibiotics resistance pattern.

Biofilm	Carbapenemase-producing strains				Antibiotic-resistant patterns														
	KPC		MBL		PIP (*n* = 19)	GEN (*n* = 82)	AMK (*n* = 33)	CIP (*n* = 89)	CAZ (*n* = 22)	CTX (*n* = 55)	IMI (*n* = 49)	MER (*n* = 44)	ERT (*n* = 47)	FOX (*n* = 100)	CFX (*n* = 20)	CPE (*n* = 24)	MDR (*n* = 45)	XDR (*n* = 21)	PDR (*n* = 10)
	POS (*n* = 52)	NEG (*n* = 57)	POS (*n* = 41)	NEG (*n* = 68)															
Strong biofilm forming (*n* = 17, 15.5%)	17 (32.6%)	0 (0%)	17 (41.4%)	1 (1.4%)	12 (63.1%)	17 (20.7%)	17 (51.1%)	17 (19.1%)	10 (45.4%)	10 (18.1%)	17 (34.6%)	17 (38.6%)	17 (36.1%)	17 (17%)	10 (50%)	17 (70.8%)	17 (37.7%)	17 (80.9%)	10 (100%)

Moderate biofilm forming (*n* = 41, 37.6%)	35 (67.3%)	6 (10.5%)	24 (58.5%)	16 (23.5%)	7 (36.8%)	41 (50%)	16 (48.4%)	41 (46.0%)	12 (54.5%)	41 (74.5%)	32 (65.3%)	27 (61.3%)	30 (63.8%)	41 (41%)	10 (50%)	8 (33.3%)	26 (57.7%)	4 (19.0%)	0 (0%)

Weak biofilm forming (*n* = 11, 10.1%)	0 (0%)	11 (19.2%)	0 (0%)	11 (16.1%)	0 (0%)	11 (13.4%)	0 (0%)	11 (12.3%)	0 (0%)	4 (7.2%)	0 (0%)	0 (0%)	0 (0%)	11 (11%)	20 (0%)	1 (4.1%)	2 (4.4%)	0 (0%)	0 (0%)

Nonbiofilm forming (*n* = 40, 36.6%)	0 (0%)	40 (70.1%)	0 (0%)	40 (58.8%)	0 (0%)	19 (23.1%)	0 (0%)	26 (29.2%)	0 (0%)	0 (0%)	0 (0%)	0 (0%)	0 (0%)	37 (37%)	0 (0%)	0 (0%)	0 (0%)	0 (0%)	0 (0%)

Clinical isolates																			
Urine (*n* = 35, 32.1%)	18 (34.6%)	17 (29.8%)	10 (24.3%)	25 (36.7%)	2 (10.5%)	24 (29.2%)	10 (30.3%)	30 (33.7%)	9 (40.9%)	15 (27.7%)	17 (34.6%)	15 (34.9%)	17 (34.6%)	30 (30%)	5 (25%)	7 (29.1%)	13 (28.8%)	4 (19.1%)	2 (20%)
Blood (*n* = 29, 26.6%)	15 (28.8%)	14 (24.5%)	20 (48.7%)	9 (13.2%)	7 (36.8%)	22 (26.8%)	5 (15.3%)	27 (30.3%)	5 (22.7%)	15 (27.7%)	13 (26.6%)	14 (31.8%)	11 (23.4%)	28 (28%)	5 (25%)	5 (20.8%)	15 (33.3%)	9 (42.2%)	4 (40%)
Wound (*n* = 19, 17.4%)	19 (36.5%)	0 (0%)	11 (26.8%)	4 (5.8%)	8 (42.1%)	19 (23.1%)	10 (30.3%)	19 (21.3%)	7 (31.8%)	19 (34.5%)	10 (20.4%)	10 (22.7%)	10 (20.4%)	19 (19%)	10 (50%)	10 (41.6%)	12 (26.6%)	7 (33.3%)	4 (40%)
Sputum (*n* = 15, 13.7%)	0 (0%)	15 (26.3%)	0 (0%)	15 (22%)	0 (0%)	10 (12.1%)	5 (15.3%)	9 (10.1%)	0 (0%)	10 (18.1%)	9 (18.3%)	5 (11.3%)	9 (18.3%)	14 (14%)	0 (0%)	2 (8.3%)	4 (8.8%)	1 (4.7%)	0 (0%)
Body fluids (*n* = 11, 10.1%)	0 (0%)	11 (31.9%)	0 (0%)	11 (16.1%)	2 (10.5%)	7 (8.5%)	3 (2.1%)	2 (2.2%)	1 (4.5%)	1 (1.8%)	0 (0%)	0 (0%)	0 (0%)	9 (9%)	0 (0%)	0 (0%)	1 (2.2%)	0 (0%)	0 (0%)

Hospital sections																			
Burn unit (*n* = 15, 13.7%)	12 (23.0%)	3 (5.2%)	9 (21.9%)	4 (6.2%)	3 (15.7%)	10 (12.1%)	9 (27.2%)	15 (16.8%)	3 (13.6%)	13 (23.6%)	10 (20.4%)	9 (20.4%)	10 (21.2%)	15 (15%)	2 (10%)	4 (16.6%)	10 (22.2%)	3 (14.2%)	3 (30%)
Emergency (*n* = 11, 10%)	7 (13.4%)	4 (7.1%)	5 (12.1%)	6 (9.3%)	0 (0%)	7 (8.2%)	3 (9.0%)	9 (10.1%)	0 (0%)	2 (3.6%)	2 (4.1%)	2 (4.5%)	2 (4.2%)	11 (11%)	1 (5%)	1 (4.1%)	2 (4.4%)	0 (0%)	0 (0%)
Internal unit (*n* = 19, 17.4%)	11 (21.1%)	7 (12.2%)	8 (19.5%)	11 (17.1%)	4 (21.0%)	11 (12.9%)	7 (21.1%)	15 (16.8%)	5 (22.7%)	9 (16.3%)	9 (18.3%)	9 (20.4%)	9 (19.1%)	19 (19%)	9 (45%)	11 (45.8%)	8 (17.7%)	4 (19.0%)	4 (40%)
Pediatrics (*n* = 9, 8.2%)	4 (7.6%)	5 (8.7%)	3 (7.3%)	5 (7.8%)	0 (0%)	9 (10.5%)	2 (6.6%)	2 (2.2%)	0 (0%)	1 (1.8%)	1 (2.0%)	1 (2.2%)	1 (2.1%)	2 (2%)	0 (0%)	0 (0%)	1 (2.2%)	0 (0%)	0 (0%)
ICU (*n* = 20, 18.3%)	7 (13.4%)	13 (22.8%)	10 (24.3%)	10 (15.6%)	5 (26.3%)	20 (23.5%)	9 (27.2%)	18 (20.2%)	6 (27.2%)	11 (20.0%)	13 (26.5%)	12 (27.2%)	11 (23.4%)	20 (20%)	6 (30%)	6 (25%)	11 (24.4%)	6 (25%)	2 (20%)
Surgery unit (*n* = 13, 11.9%)	10 (19.2%)	3 (5.2%)	5 (12.1%)	8 (12.5%)	7 (36.8%)	13 (15.2%)	3 (9.9%)	10 (11.2%)	9 (40.9%)	13 (23.6%)	10 (20.4%)	7 (15.9%)	10 (21.2%)	11 (11%)	2 (10%)	2 (8.3%)	9 20.0%)	9 (42.8%)	1 (10%)
Nephrology (*n* = 22, 20.8%)	1 (1.9%)	21 (36.8%)	1 (2.4%)	21 (32.8%)	0 (0%)	12 (14.1%)	0 (0.0%)	20 (22.4%)	0 (0%)	5 (90%)	4 (18.1%)	4 (9.1%)	4 (8.5%)	22 (22%)	0 (0%)	0 (0%)	4 (8.8%)	0 (0%)	0 (0%)

OXA genes																			
OXA-145 (*n* = 20, 18.3%)	17 (32.6%)	3 (5.2%)	20 (48.7%)	0 (0%)	4 (21.0%)	20 (24.3%)	9 (27.1%)	20 (22.4%)	19 (83.6%)	20 (36.6%)	20 (40.8%)	20 (45.4%)	20 (42.5%)	20 (20%)	17 (85%)	13 (54.1%)	20 (44.4%)	21 (100%)	10 (100%)
OXA-224 (*n* = 24, 22.0%)	20 (38.4%)	4 (7.0%)	23 (56.0%)	1 (1.5%)	3 (15.7%)	24 (29.2%)	10 (30.3%)	24 (26.9%)	22 (100%)	24 (43.6%)	24 (48.9%)	24 (54.5%)	24 (51.0%)	24 (24%)	20 (100%)	24 (100%)	24 (43.3%)	21 (100%)	10 (100%)
OXA-539 (*n* = 41, 40.3%)	41 (78.8%)	0 (0%)	41 (100%)	0 (31.9%)	11 (57.8%)	41 (50%)	13 (39.3%)	41 (46.0%)	22 (100%)	41 (74.5%)	41 (83.6%)	41 (93.1%)	41 (87.2%)	41 (41%)	20 (100%)	24 (100%)	41 (91.1%)	21 (100%)	10 (100%)
OXA-675 (*n* = 11, 10.0%)	9 (17.3%)	2 (3.5%)	11 (26.8%)	0 (0%)	1 (5.2%)	11 (21.1%)	1 (3.0%)	11 (12.3%)	10 (47.6%)	11 (20%)	11 (22.4%)	11 (25%)	11 (23.4%)	11 (11%)	6 (30%)	2 (8.3%)	11 (24.4%)	10 (47.6%)	10 (100%)

PIP: piperacillin; GEN: gentamycin; AMK: amikacin; CIP: ciprofloxacin; CAZ: ceftazidime; CTX: ceftriaxone; IMI: imipenem; MER: meropenem; ERT: ertapenem; FOX: cefoxitin; CFX: cefazolin; CPE: cefepime; MDR: multiple drug resistance; XDR: extensively drug-resistant; PDR: pan drug-resistant; ICU: intensive care unit.

**Table 3:** Correlation between OXA genes, biofilm formation, separated clinical isolates, and antibiotic resistance in *P. aeruginosa*.

Biofilm	OXA genes					
	blaOXA-145	blaOXA-161	blaOXA-224	blaOXA-539	blaOXA-675	blaOXA-848
Strong	*P* = 0.009	*P* = 0.001	*P* = 0.008	*P* = 0.009	*P* = 0.039	*P* = 0.022
Moderate	*P* = 0.017	*P* = 0.050	*P* = 0.036	*P* = 0.002	*P* = 0.075	*P* = 0.033
Weak	*P* = 0.043	*P* = 0.003	*P* = 0.032	*P* = 0.017	*P* = 0.015	*P* = 0.048
Antibiotic resistance						
CEF	*P* = 0.040	*P* = 0.029	*P* = 0.016	*P* = 0.048	*P* = 0.066	*P* = 0.043
PIP	*P* = 0.671	*P* = 0.371	*P* = 0.40	*P* = 0.11	*P* = 0.96	*P* = 0.115
GEN	*P* = 0.250	*P* = 0.089	*P* = 0.014	*P* = 0.097	*P* = 0.25	*P* = 0.01
AMK	*P* = 0.111	*P* = 0.089	*P* = 0.077	*P* = 0.091	*P* = 0.088	*P* = 0.049
NOR	*P* = 0.058	*P* = 0.060	*P* = 0.084	*P* = 0.12	*P* = 0.09	*P* = 0.049
CIP	*P* = 0.31	*P* = 0.11	*P* = 0.33	*P* = 0.47	*P* = 0.075	*P* = 0.082
CAZ	*P* = 0.085	*P* = 0.020	*P* = 0.009	*P* = 0.005	*P* = 0.066	*P* = 0.043
CTX	*P* = 0.072	*P* = 0.061	*P* = 0.049	*P* = 0.021	*P* = 0.013	*P* = 0.069
IMI	*P* = 0.049	*P* = 0.016	*P* = 0.055	*P* = 0.005	*P* = 0.009	*P* = 0.045
MER	*P* = 0.019	*P* = 0.062	*P* = 0.035	*P* = 0.015	*P* = 0.009	*P* = 0.45
ERT	*P* = 0.31	*P* = 0.11	*P* = 0.33	*P* = 0.47	*P* = 0.075	*P* = 0.082
FOX	*P* = 0.025	*P* = 0.015	*P* = 0.084	*P* = 0.001	*P* = 0.075	*P* = 0.033
CFX	*P* = 0.002	*P* = 0.001	*P* = 0.072	*P* = 0.050	*P* = 0.039	*P* = 0.044
MBL	*P* = 0.019	*P* = 0.020	*P* = 0.048	*P* = 0.053	*P* = 0.036	*P* = 0.050
KPC	*P* = 0.094	*P* = 0.31	*P* = 0.19	*P* = 0.020	*P* = 0.62	*P* = 0.27
MDR	*P* = 0.017	*P* = 0.039	*P* = 0.047	*P* = 0.050	*P* = 0.038	*P* = 0.019
XDR	*P* = 0.049	*P* = 0.046	*P* = 0.055	*P* = 0.025	*P* = 0.019	*P* = 0.065
Clinical specimens						
Urine	*P* = 0.029	*P* = 0.036	*P* = 0.015	*P* = 0.077	*P* = 0.059	*P* = 0.045
Blood	*P* = 0.049	*P* = 0.016	*P* = 0.055	*P* = 0.012	*P* = 0.019	*P* = 0.016
Wound	*P* = 0.017	*P* = 0.022	*P* = 0.034	*P* = 0.019	*P* = 0.049	*P* = 0.011
Sputum	*P* = 0.087	*P* = 0.092	*P* = 0.096	*P* = 0.190	*P* = 0.130	*P* = 0.220
Body fluids	*P* = 0.120	*P* = 0.151	*P* = 0.192	*P* = 0.099	*P* = 0.101	*P* = 0.100

## Data Availability

The datasets used and/or analyzed during the current study are available from the corresponding author upon reasonable request.
